# Distinguishing Planting Structures of Different Complexity from UAV Multispectral Images

**DOI:** 10.3390/s21061994

**Published:** 2021-03-12

**Authors:** Qian Ma, Wenting Han, Shenjin Huang, Shide Dong, Guang Li, Haipeng Chen

**Affiliations:** 1 Institute of Soil and Water Conservation, Chinese Academy of Sciences, Ministry of Water Resources, Yangling 712100, China; maqian18@mails.ucas.ac.cn; 2 College of Advanced Agricultural Sciences, University of Chinese Academy of Sciences, Beijing 100049, China; 3 College of Mechanical and Electronic Engineering, Northwest A&F University, Yangling 712100, China; ShenjinHuang@nwafu.edu.cn (S.H.); liguang@nwafu.edu.cn (G.L.); haipengchen@nwafu.edu.cn (H.C.); 4 Institute of Geographic Sciences and Natural Resources Research, Chinese Academy of Sciences, Beijing 100101, China; dongsd.17b@igsnrr.ac.cn

**Keywords:** UAV, multispectral remote sensing, farmland objects, classification, RF, SVM

## Abstract

This study explores the classification potential of a multispectral classification model for farmland with planting structures of different complexity. Unmanned aerial vehicle (UAV) remote sensing technology is used to obtain multispectral images of three study areas with low-, medium-, and high-complexity planting structures, containing three, five, and eight types of crops, respectively. The feature subsets of three study areas are selected by recursive feature elimination (RFE). Object-oriented random forest (OB-RF) and object-oriented support vector machine (OB-SVM) classification models are established for the three study areas. After training the models with the feature subsets, the classification results are evaluated using a confusion matrix. The OB-RF and OB-SVM models’ classification accuracies are 97.09% and 99.13%, respectively, for the low-complexity planting structure. The equivalent values are 92.61% and 99.08% for the medium-complexity planting structure and 88.99% and 97.21% for the high-complexity planting structure. For farmland with fragmentary plots and a high-complexity planting structure, as the planting structure complexity changed from low to high, both models’ overall accuracy levels decreased. The overall accuracy of the OB-RF model decreased by 8.1%, and that of the OB-SVM model only decreased by 1.92%. OB-SVM achieves an overall classification accuracy of 97.21%, and a single-crop extraction accuracy of at least 85.65%. Therefore, UAV multispectral remote sensing can be used for classification applications in highly complex planting structures.

## 1. Introduction

According to statistics published by the United Nations, the world population is expected to reach about 10 billion in 2050 [1,2]. Population expansion brings new challenges to the maintenance of food production security. Mastering the area and spatial distribution of regional crops is the prerequisite for accurately obtaining regional crop yields, and the rational allocation of regional water resources. However, smallholders or family farms, which are still prevailing in some developing countries, are responsible for a large share of the world food production. The scattered farmland and discrete crops of smallholders make cropland mapping and monitoring more difficult, affecting the accurate estimation of regional crop yields and the rational allocation of water resources. The emergence of remote sensing technology has promoted agricultural production and research, from the traditional stage to the stage of refinement, quantification, and mechanism. High-quality remote sensing images, especially those of high resolution, can extract feature information from the ground, making the fine classification and monitoring of ground details possible [3,4]. Agricultural information at the farmland scale can be directly applied to the optimization of cultivation management and the analysis of breeding decisions. It has further applications compared with the large-scale agricultural monitoring technology used for macro decision-making [5]. Moreover, the acquisition of farmland-scale agricultural information places more stringent requirements on image data timeliness and spatial resolution. At present, the monitoring platform for acquiring crop planting information on the farmland scale is mainly based on high-resolution satellite remote sensing and unmanned aerial vehicle (UAV) remote sensing.

Compared with satellite remote sensing, UAV remote sensing is less expensive, offers more flexible mobility, and has a short operating cycle, thus providing several advantages in precision agriculture [6,7,8,9]. Researchers have focused on the extraction and classification of single and multiple farmland features, based on UAV remote sensing technology and remote sensing recognition algorithms, in recent years. Supervised classification is widely used to achieve high-precision classification results. There are many supervised classification algorithms, such as the maximum likelihood, single linkage, Mahalanobis distance, support vector machines and random forests. Among them, support vector machines (SVMs) and random forests (RFs) have been widely used in recent years, as they typically offer superior classification [10,11,12,13]. Compared with traditional statistical theory, SVMs with simple structures and strong generalization ability can solve a large number of small-sample learning problems [14]. RF uses sample disturbance and attributes disturbance to achieve good robustness in classification convergence and generalization error [15]. Besides this, deep learning, as an extension of the artificial neural network method, is a new exciting and effective classification method. However, its high requirement of samples increases its cost and limits its application in areas lacking samples [16,17].

The extraction of single-crop planting information is mainly realized by appropriate remote sensing recognition algorithms based on the unique phenological characteristics of a crop. At present, the extraction of large-scale crops, such as corn, wheat, rice, tobacco, and fruit trees, is based on pixel- and object-oriented classification algorithms, and the classification accuracy can reach more than 90% [18,19,20,21]. Liu et al. [22] reported a classification accuracy of winter wheat planting information that exceeded 93.0%, using a deep learning algorithm for pixels combined with UAV visible light remote sensing data. Hall et al. [23] constructed spectral and texture features for maize information from UAV multispectral remote sensing data, and applied an object-oriented SVM to realize the extraction of corn planting information with an extraction accuracy of 94.0%. However, in the case of mixed crops or the intercropping of multiple crops, the fine extraction of multi-crop information suffers significant interference. It requires more robust recognition algorithms than single-crop information extraction. Object-oriented image analysis (OBIA) takes the speckle as the primary processing unit. It considers the spectrum, texture, and context as input features, effectively solving the “salt and pepper” phenomenon in pixel classification [24,25]. Scholars have used OBIA to achieve good classification results for complex planting structures containing between three and five different crops [26,27,28,29]. For example, based on UAV images, OBIA was used to classify five types of crops with an overall accuracy of 84.7% [30]. Nevertheless, the extraction accuracy of multiple crops is still insufficient because the similarity of two or more crops is high. Wu et al. [31] used a portable UAV to obtain light images, and used an object-oriented classification method to classify rice, lotus root, and vegetables. Their results showed that the mixed classification of lotus root and vegetable land presented a severe challenge, with a relative error as high as 193%. Liu et al. [32] used SVM to distinguish corn, wheat, trees, greenhouses, and other ground features, based on UAV visible images data and digital surface model (DSM) data. Their results indicated that using only spectral features for classification would lead to confusion between trees and corn.

In general, the information extraction of single farmland features based on UAV remote sensing data is relatively mature, and the extraction accuracy is high. However, there is still some confusion in the classification of many kinds of crops. In addition, multiple crop classification mainly focuses on three to five different crops, and there have been few comparative studies in which the cropping structures have different levels of complexity. Therefore, this paper describes the use of UAV remote sensing technology to classify farmland features in study areas with different levels of planting structure complexity. The aims of this study are as follows: (1) explore the applicability of UAV multispectral remote sensing recognition algorithms for farmland feature classification with planting structures that have different degrees of complexity; and (2) analyze the potential for UAV multispectral remote sensing technology to be used for complex planting structure extraction.

## 2. Study Area and Data Preparation

### 2.1. Overview of the Study Area

**Figure 1:**
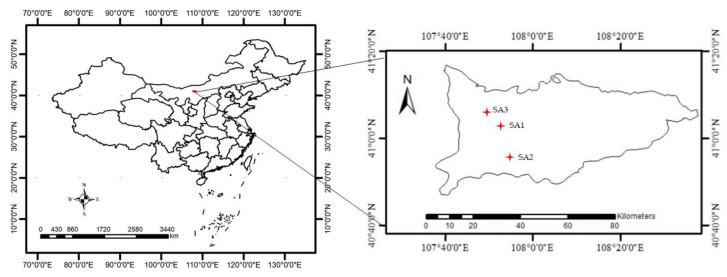
Geographical location of the study areas.

The study areas are located in Wuyuan County, part of the Inner Mongolia Autonomous Region of China, which have a typical mid-temperate continental monsoon climate. The geographic location map of the study areas is shown in Figure 1. The study areas are arid and receives plenty of sunshine. The annual rainfall is only 130–285 mm, and the annual total amount of solar radiation is as high as 6424.23 MJ·m^−2^. The rich water resources in these area benefit from the Yellow River diversion irrigation system, and can completely satisfy the needs of local crops. This study considers three areas in Wuyuan County. Study area 1 (SA1) is in Taerhu (49.99°N, 107.83°E), study area 2 (SA2) is in Fuxing (41.12°N, 107.96°E), and study area 3 (SA3) is located in Yindingtu (41.18°N, 107.84°E). SA1 contains three types of crops (corn, sunflower, and zucchini), and is selected as a district with low planting structure complexity. SA2 contains five types of crops (corn, sunflower, zucchini, hami melon, and pepper), and is selected as a district with medium planting structure complexity. SA3 contains eight types of crops (sunflower, zucchini, hami melon, pepper, sapling, watermelon, cherry tomato, and tomato), and is selected as a district with high planting structure complexity. During the experimental period, the corn was in the jointing stage, the sunflower was in the budding stage, and the zucchini, hami melon, pepper, watermelon, cherry tomato, and tomato were all in the fruiting stage.

### 2.2. The Collection of UAV Remote Sensing Data

An information collection system based on a UAV (S900, DJI Technology Co., Shenzhen, China) was adopted to collect the multispectral remote sensing images. The system integrated UAV flight control and the corresponding position and orientation system (POS) data acquisition. It could stably obtain UAV multispectral images without distortion. The multispectral camera (MicaSense RedEdge-M, MicaSense, Seattle, WA, USA) could obtain red, green, blue, near-infrared, and red edge band data. Detailed information of the UAV and multispectral camera is presented in Table 1.

The spectral characteristics of crops vary significantly under different phenological periods and light conditions. The UAV remote sensing tests were conducted on 26, 29 July and 1 August 2020, which had similar meteorological conditions. The meteorological data obtained from the local weather bureau were the average values from 11 a.m. to 2 p.m. during the test period (shown in Table 2). The three experimental days were sunny days with lower wind speed, fewer air pollutants, and higher illuminance, all suitable for UAV flight operations.

**Figure 2:**
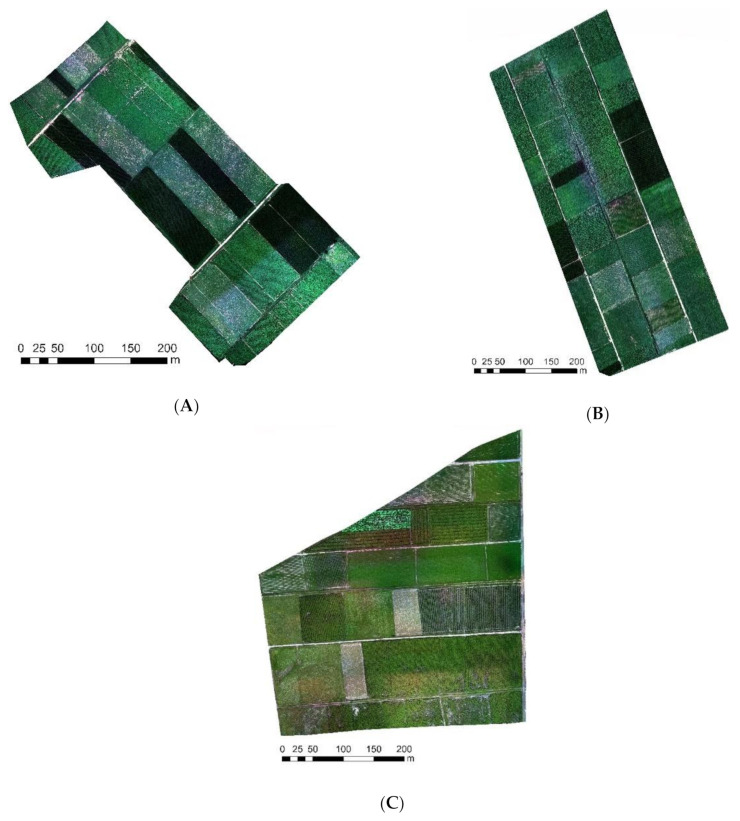
UAV images mosaic results of the study areas.((**A**) UAV images mosaic result of study area 1; (**B**) UAV images mosaic result of study area 2; (**C**) UAV images mosaic result of study area 3).

The flight altitude was set to 100 m above the ground, the course overlap was 70%, and the horizontal overlap was 65%. The RAW format images were exported and converted to TIFF format using the PixelWrench2 software installed in the camera. The spectral reflectivity was calculated using ENVI (v. 5.1, Exelis Visual Information Solutions, Boulder, CO, USA) combined with the standard whiteboard data. The ground control points (GCPs) are vital in verifying the accuracy of terrain information obtained by UAV. The 3D coordinates of the GCPs in this study were accurately measured by a real-time kinematic (RTK) product (Zhonghui i50, CHCNAV, Shanghai, China), which has a high precision of 2 mm. According to the actual terrain conditions and the control point layout principle, six GCPs were selected in each study area. Among them, three base numbers were used as calibration points and three even numbers were used as check points. The control points were set at the intersections of hardened roads. They were easy to distinguish and had good stability. Images were stitched using Pix4DMapper (v. 3.2, Pix4D, Prilly, Switzerland) based on the TIFF multispectral images and POS data collected by the UAV remote sensing system. In this study, 1540 multispectral remote sensing images were obtained from the three study areas. The data contained grayscale information such as red, blue, green, near-infrared, and red edge bands, and the spatial resolution was 7 mm. The UAV images’ mosaic results of the study areas are shown in Figure 2.

### 2.3. The Collection of Ground Data

During the experiment, we collected the ground distribution data and ground spectral data of crops. The ground distribution data of crops is the basis for selecting training samples and verification samples. This can help to evaluate the classification results visually. Ground spectral data of crops can help us explore the differences in crop spectral characteristics better, provide a theoretical basis for the classification results, and analyze the error sources in the classification results effectively.

#### 2.3.1. The Ground Distribution Data of Crops

**Figure 3:**
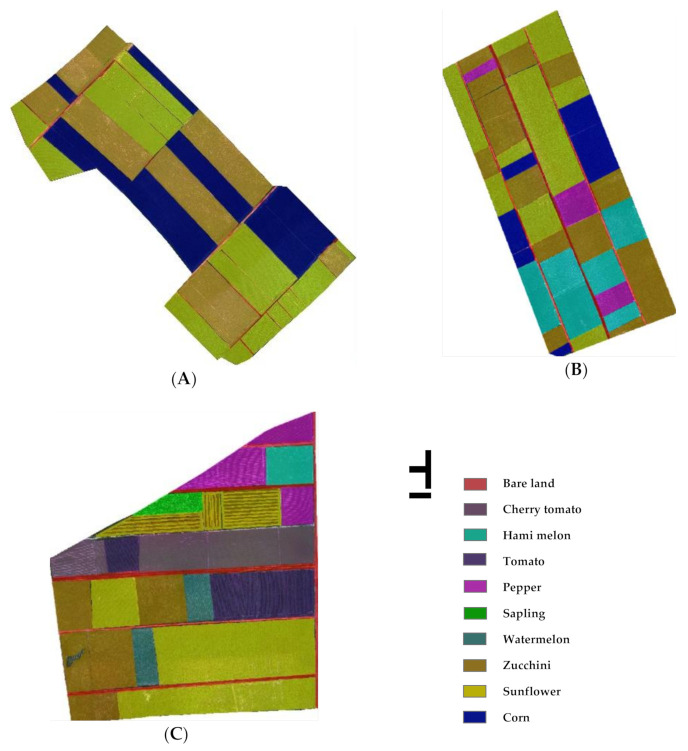
The ground crop distribution maps. ((**A**) the standard ground crop distribution map of study area 1; (**B**) the ground crop distribution map of study area 2; (**C**) the ground crop distribution map of study area 3).

The types of crops were determined based on field surveys, and the location of each crop was recorded using portable RTK in units of plots. Combining ground data and UAV images, the ground crops distribution maps (Figure 3) were drawn.

#### 2.3.2. Crop–Ground Spectral Curves

**Figure 4:**
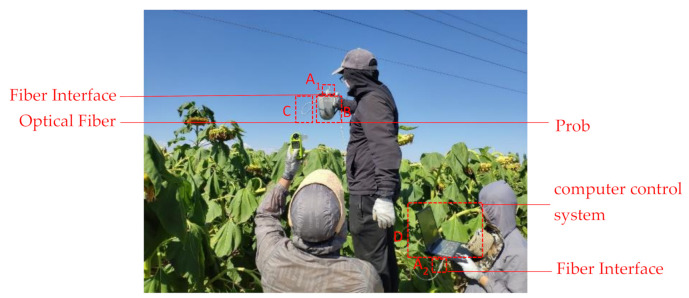
Schematic diagram of spectral curve collection work.

The crop–ground spectral curves in this study were obtained by FieldSpec Hand Held (ASD, Westborough, CO, USA) on a sunny day (1 August, 11.00–14.00). The specific parameters of the FieldSpec Hand Held are shown in Table 3. As shown in Figure 4**,** the field experimenters had to wear dark clothes and face the sun when collecting ground spectral data. First, the optical fiber probe was aligned at the whiteboard for correction, and then aligned at the vegetation canopy to collect reflectance spectra. Six samples were randomly selected from each type of crop, and ten spectral curves were measured for each plant sample, which was arithmetically averaged to obtain the final reflectance spectral data of the sample.

## 3. Research Procedure and Method

**Figure 5:**
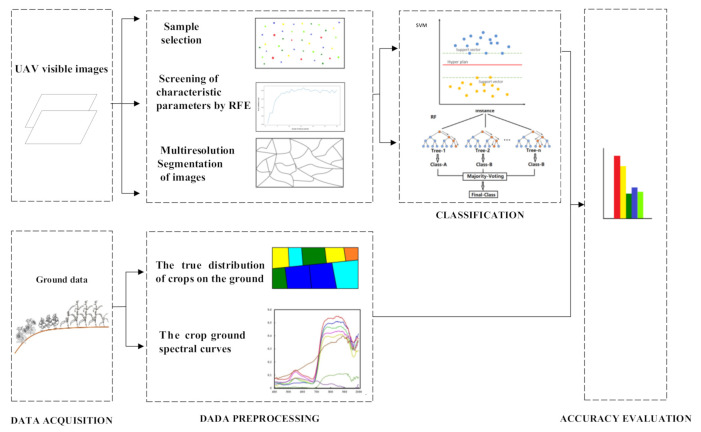
Workflow of planting structure extraction.

The workflow of planting structure extraction is shown in Figure 5. There are seven main stages: (1) the acquisition and preprocessing of UAV remote sensing images, including the construction of the UAV multispectral system, the selection of an aerial photography path, and the stitching and geometric correction of orthophoto images; (2) the collection of ground data, including the investigation of the true distribution of crops on the ground and the collection of crop–ground spectral curves; (3) the selection of training and verification samples of UAV multispectral images; (4) multiscale segmentation of UAV images; (5) the extraction of features and the determination of feature subsets, including the extraction of spectral features and texture features, and the selection of the best feature band based on recursive feature elimination (RFE); (6) the use of object-oriented RF (OB-RF) and object-oriented SVM (OB-SVM) classification models to classify farmland crops; (7) the use of confusion matrices to evaluate and compare the classification accuracy of each model.

### 3.1. Sample Selection

The types of crops in the three study areas were determined through field research, and RTK was used to calibrate each crop’s geographic location. We randomly generated samples based on the ground standard crop distribution maps (Figure 3). In three study areas, the reference samples were randomly split into two sets of disjointed training samples (TS) and validation samples (VS), via the sample-function in R (v. 4.0.3). The selection results of the samples are shown in Table 4.

### 3.2. Construction and Screening of Feature Parameters

#### 3.2.1. Construction of Spectral Features and Texture Features

Vegetation indices can magnify the spectral information between ground objects, and are one of the simplest and most effective methods of studying vegetation characteristics. In this study, eight common vegetation indices were obtained from band calculations (shown in Table 5): the normalized difference vegetation index (NDVI) [33], the ratio vegetation index (RVI) [34], the difference vegetation index (DVI) [35], excess green (EXG) [36], the visible-band difference vegetation index (VDVI) [37], the normalized green–blue difference index (NGBDI) [38], the normalized green–red difference index (NGRDI) [39], and the Woebbecke index (WI) [40]. Texture features can reflect the characteristics of homogeneity in the images, and are unaffected by image color and image brightness The common texture features include the mean, variance, synergy, contrast, dissimilarity, information entropy, second moment, and correlation. This study obtained 40 texture features of crops in five bands (red, green, blue, near-infrared, red edge) by applying co-occurrence measures, which calculated texture values using the grey tone spatial dependence matrix. This process was implemented in ENVI 5.1 (Exelis Visual Information Solutions, Boulder, CO, USA). In addition, the size of the filtering window was 3×3, which was the default value in ENVI.

#### 3.2.2. Screening of Characteristic Parameters

To improve the operation speed and prediction accuracy of the model and avoid overfitting, the feature parameters of the images were screened to eliminate features that had a low correlation with the model prediction. Recursive feature elimination (RFE) is an efficient algorithm that combines classifiers to find the optimal feature subset [41]. It creates the model repeatedly, and retains the best features or removes the worst features in each iteration. In subsequent iterations, it uses features that were not selected in the previous model to create the next model until all features are exhausted. Finally, RFE ranks the features according to the order in which they were retained or removed, and selects the best subset. This study performed feature optimization based on the RFE module in scikit-learn, a free software machine learning library basing the Python programming language. The RF classifier was used to evaluate the RFE model, and ten-fold cross-validation was adopted to evaluate the model parameters’ accuracy.

**Figure A1:**
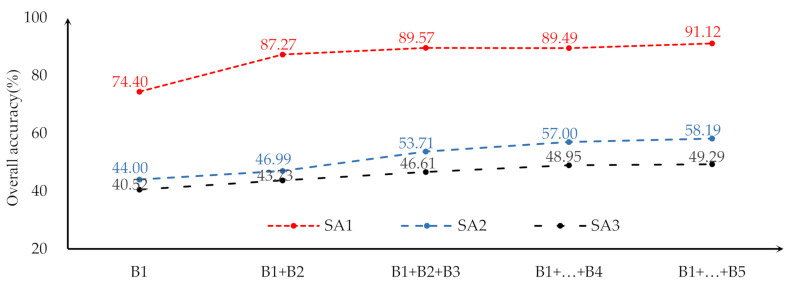
Relationship between accumulated multispectral bands and overall accuracy.

**Figure A2:**
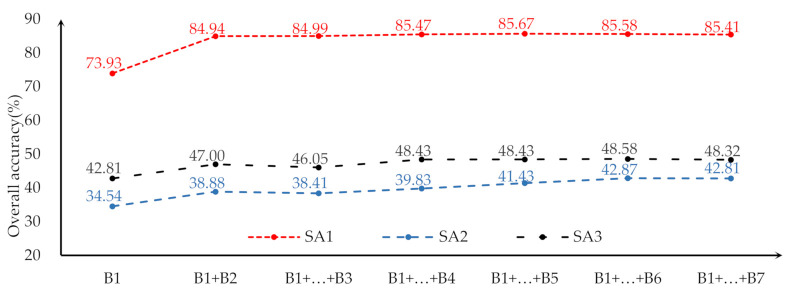
Relationship between accumulated vegetation indices and overall accuracy.

**Figure A3:**
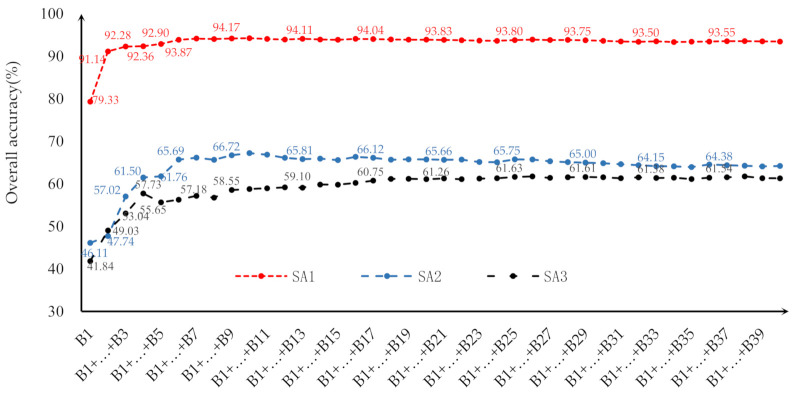
Relationship between accumulated textural features and overall accuracy.

RFE was used to screen features in five spectral bands, seven vegetation indices, and 40 texture features. The importance rankings of the features are shown in Table A1, Table A2 and Table A3. The characteristics ranked first, second, third (and so on) in their corresponding feature sets were denoted as B1, B2, B3 (and so on). The feature parameters were accumulated one by one as per the importance rankings of the feature parameters, and the images were pre-classified based on the accumulated feature subset. The classification accuracy is shown in Figure A1, Figure A2 and Figure A3. According to the importance rankings of all features and the pre-classification results in Figure A1, Figure A2 and Figure A3, the feature subset was then constructed by retaining the features that contributed significantly to the classification, and eliminating the features that contributed little or were useless. The final filtering results are presented in Table 6.

### 3.3. Multiresolution Segmentation

**Figure 6:**
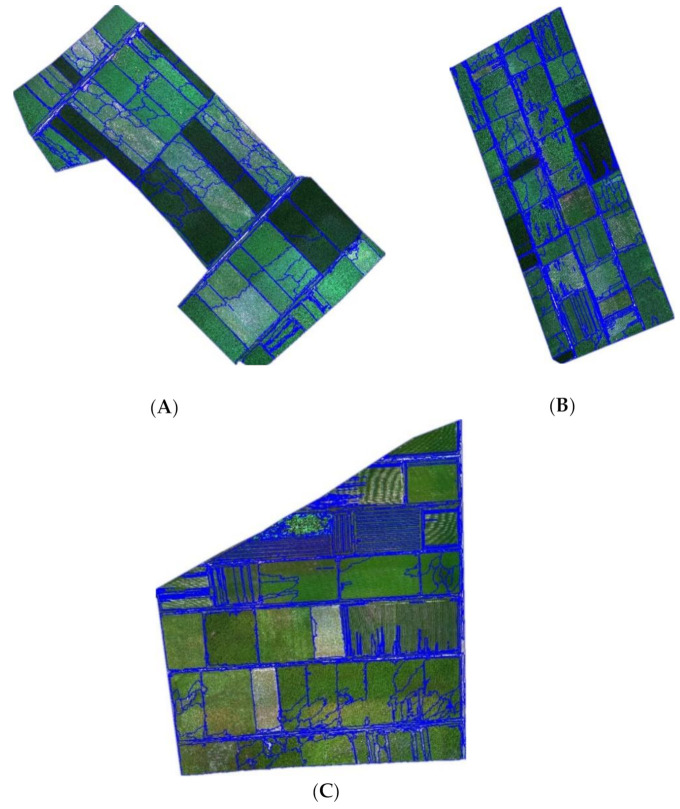
Image segmentation results of the study areas. ((**A**) image segmentation result of study area 1; (**B**) image segmentation result of study area 2; (**C**) image segmentation result of study area 3).

OBIA makes full use of the spatial, textural, contextual, and other geometric features and structure information of remote sensing images. It is superior to pixel-oriented analysis for crop extraction because it efficiently solves the problems of “same substance with a different spectrum”, “same spectrum with a foreign substance” and the “salt and pepper effect” [24]. OBIA uses an iterative algorithm to segment remote sensing images into uniform and continuous image objects. OBIA mainly has two independent modules: object generation and image information extraction. A good segmentation effect is the prerequisite to achieving excellent classification results [25]. Generally, the ground feature information is complex and mixed, making it challenging to obtain an ideal segmentation effect using a single-scale segmentation method. Therefore, multiresolution segmentation is commonly adopted for land use information extraction. This method creates image polygon objects with arbitrary scales and similar attribute information. Through multiresolution segmentation, adjacent similar pixels gather to form objects, and the classifier uses these homogeneous objects as the basic processing units to extract information from images. In this study, remote sensing images were first segmented into image objects with different scales, based on the multiscale segmentation method. Then, target crop extraction was accomplished using spectral and textural features of the objects. The data processing was carried out by eCognition Developer (v. 9.2.1, Trimble Geospatial). The segmentation parameters were adjusted through multiple segmentation experiments based on expert knowledge. The principle of hyper-parameter selection was that the segmentation effect best fits the ridge line. The segmentation parameters were adjusted many times to ensure optimal values during the multiscale segmentation. The optimal segmentation parameters for remote sensing images were determined to be as follows: the segmentation scale was set to 200, the shape weight was set to 0.2, and the compactness weight was set to 0.5 through segmentation experiments. The final segmentation results are shown in Figure 6.

### 3.4. Classification Methods

#### 3.4.1. RF

RF is a nonparametric machine learning algorithm composed of multiple decision trees. This algorithm has high prediction accuracy, good tolerance to outliers and noise, a wide range of applications, and cannot easily be overfitted [42]. According to statistical learning theory, RF uses the bootstrap resampling method to extract multiple samples from the original data, and then performs decision tree modeling for each sample. The prediction results from various decision trees are synthesized, and finally, a random forest with a mass of classification trees is constructed [43]. Two parameters need to be defined to generate prediction models: the number of expected classification trees (ntree) and the number of features extracted when nodes are split (mtry). The implementation of the RF model in this study was based on the Random Forest module in scikit-learn, based on the Python programming language. It was found that setting the ntree to 50 produced an error that gradually converged and tended to be stable, while mtry was set to the square root of the total number of features.

#### 3.4.2. SVM

SVM is based on the Vapnik–Chervonenkis dimension theory of statistical learning and the principle of minimum structural risk. It is often used to solve small-sample, nonlinear, and high-dimensional pattern recognition problems [44]. Under the condition of limited sample information, SVM provides a good balance between the complexity and learning of the model, and has a good generalization ability. The common kernel functions in the SVM algorithm are linear, polynomial, radial basis, and sigmoid kernel functions. The radial basis kernel function is the most widely used as it has fewer parameters and better performance than the others, regardless of the number of samples [45]. The implementation of the SVM model in this study was based on the support vector machines module in scikit-learn based on the Python programming language.

### 3.5. Classification Accuracy Assessment

Based on the verification sample data, a confusion matrix was used to calculate the user accuracy (UA), production accuracy (PA), extraction accuracy (F), overall accuracy (OA), and Kappa coefficient. UA and PA can be used to evaluate misclassification and omission errors quantitatively, and the overall accuracy and the Kappa coefficient (K) are commonly used to evaluate the overall classification effect. Besides this, F is used to evaluate the extraction accuracy of all kinds of ground objects under various methods.

$$P_{0}=T_{c}/A_{c}$$

where *P*_0_ represents the overall classification accuracy, *T*_c_ represents the number of pixels correctly classified by method *c*, and *A*_c_ represents the total number of pixels classified by method *c*.

$$K\;=\;\frac{P_{o}\;-\;P_{e}}{1\;-\;P_{e}},\;P_{e}\;=\;\frac{a_{1}\;\times \;b_{1}\;+\;a_{2}\;\times \;b_{2}\;+\cdots +\;a_{c}\;\times \;b_{c}}{n\;\times \;n}$$

where *P*_0_ represents the overall classification accuracy, assuming that the true number of samples of each category is $a_{1}$, $a_{2}$, ⋯, $a_{c}$, the predicted number of samples of each category is $b_{1}$, $b_{2}$, ⋯, $b_{c}$, and the total number of samples is *n*.

$$F\;=\;\frac{2P_{Am}U_{Am}}{P_{Am}\;+\;U_{Am}}\;\times \;100\%$$

where *F* represents the extraction precision, *P*_Am_ represents the production accuracy of category *m*, and *U*_Am_ represents the user precision of category *m*.

## 4. Results

**Figure 7:**
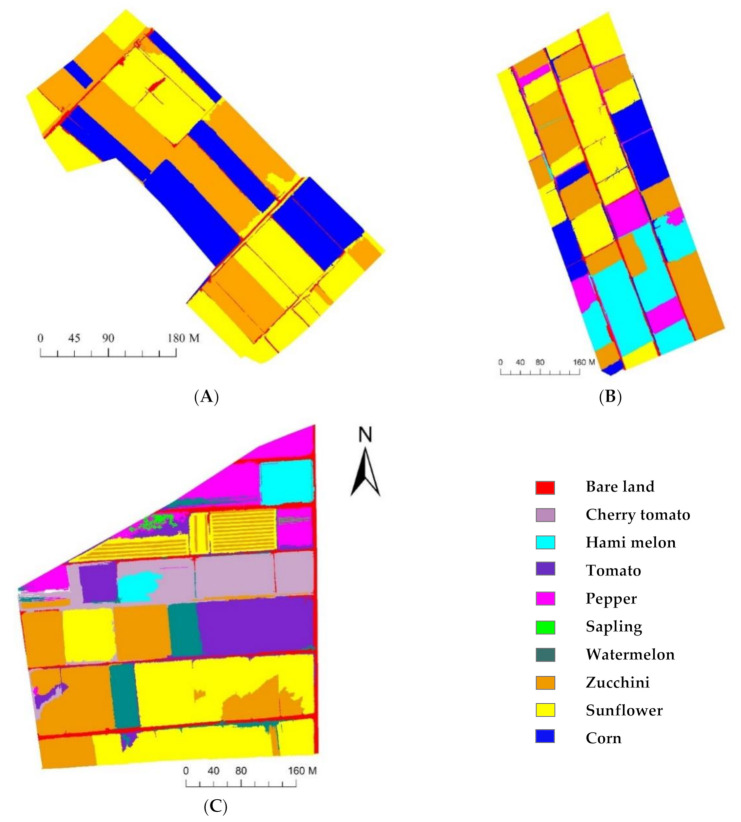
Classification results of OB-RF model. ((**A**) classification result of OB-RF model in study area 1; (**B**) classificaTable 2. (**C**) classification result of OB-RF model in study area 3). **Note:** OB-RF stands for Object-oriented random forest classification model.

**Figure 8:**
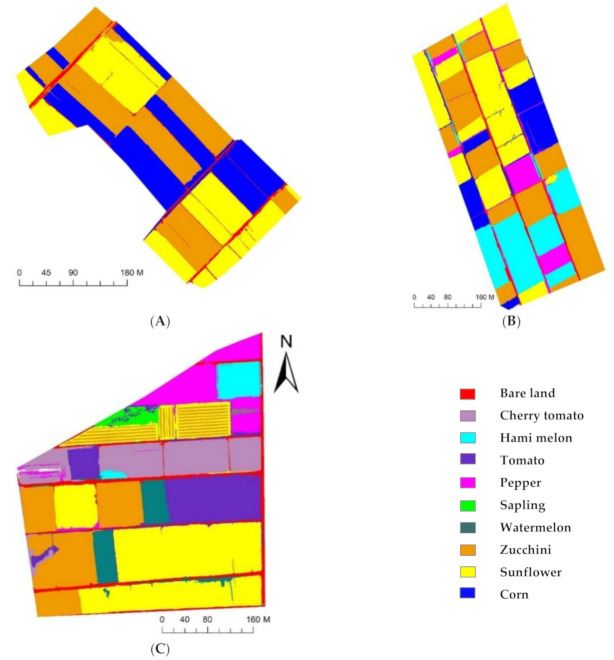
Classification results of OB-SVM model. ((**A**) classification result of OB-SVM model in study area 1; (**B**), classification result of OB-SVM model in study area 2; (**C**) classification result of OB-SVM model in study area 3). **Note:** OB-SVM stands for support vector machine classification model.

The crop planting information in three study areas with different planting complexities was extracted using OB-RF and OB-SVM (Figure 7 and Figure 8), based on the multispectral remote sensing images obtained by the UAV in the three study areas. The confusion matrix was used to evaluate the accuracy of the classification results. It assumed that pixels at the reference locations could be assigned to single classes, and accuracy measures based on the proportion of area correctly classified were then calculated from the number of correctly classified pixels [46]. The accuracy evaluation results are presented in Table 7, Table 8 and Table 9. In SA1, both OB-RF and OB-SVM achieved good classification results, with an overall accuracy greater than 97% and an extraction accuracy for every crop greater than 92%. The accuracy of SA2 was slightly lower, but the overall accuracy was still above 92%. The extraction accuracy of the OB-RF model for pepper and hami melon was low (84.86% for pepper, 75.65% for Hami melon), while the extraction accuracy of the OB-SVM model for all crops remained at a high level (extraction accuracy greater than 94%). In SA3, the overall accuracy and extraction accuracy based on the OB-SVM model remained high (overall accuracy of 97.21%, extraction accuracy greater than 85.65%). However, the overall accuracy and extraction accuracy given by the OB-RF model decreased significantly. Among all study areas, corn had the highest extraction accuracy, and saplings had the lowest extraction accuracy.

## 5. Discussion

### 5.1. Classification Error Analysis

**Figure 9:**
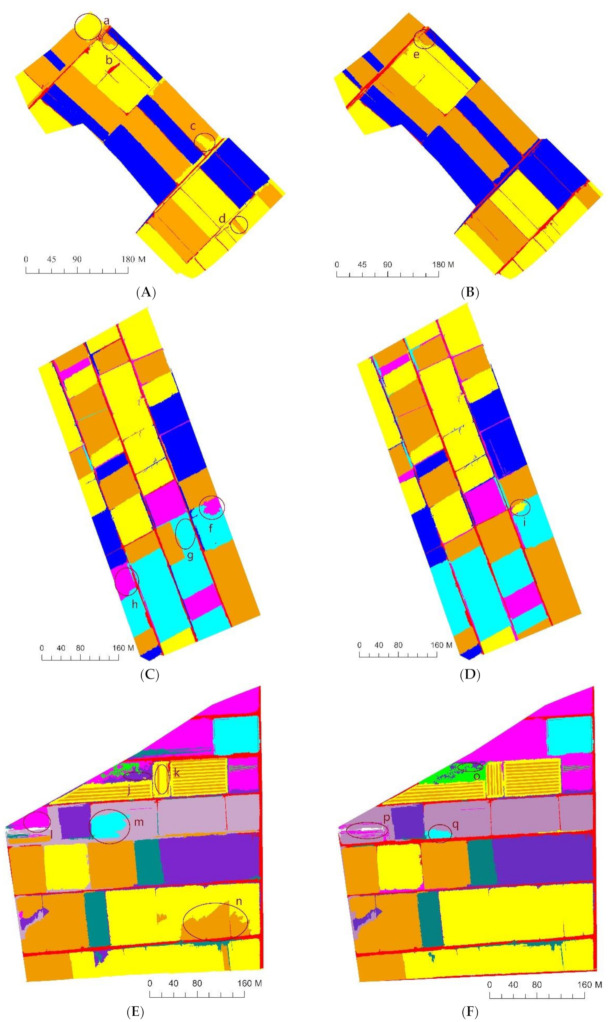
Classification error map of OB-RF and OB-SVM in study areas. ((**A**) classification error map of OB-RF in study area 1; (**B**) classification error map of OB-SVM in study area 1; (**C**) classification error map of OB-RF in study area 2; (**D**) classification error map of OB-SVM in study area 2; (**E**), classification error map of OB-RF in study area 3; (**F**) classification error map of OB-SVM in study area 3.)

By comparing the classification results obtained by OB-SVM and OB-RF (Figure 7 and Figure 8) with the standard crops distribution map obtained through field investigation (Figure 3), classification error detail maps (Figure 9) were made. In SA1, the primary source of error was the mixed classification of zucchini and sunflower. In SA2, the primary source of error was the mixed fraction of cantaloupe and zucchini, and the mixed fraction of pepper and cantaloupe. In SA3, the primary source of error was the mixed fraction of hami melon and cherry tomato, the mixed fraction of pepper and cherry tomato, and the mixed fraction of zucchini and sunflower. In general, there are mainly five crops that are easy to mix: hami melon, pepper, zucchini, cherry tomato, and sunflower. In order to explore the reasons for crop mixing, we analyzed the spectral curves of five easily mixed crops. The spectral curves of five crops with a high mixing frequency (sunflower, cherry tomato, pepper, hami melon, and zucchini) are shown in Figure 9. In the spectral range 400–900 nm, the spectral reflectance of five easily confused crops is stable in the near-infrared band range from 770 to 800 nm, where the difference is most apparent. Additionally, there are apparent reflection peaks in the green band from 540 to 560 nm, and some differences in the height of reflection peaks of different crops. However, for both 770–800 nm and 540–560 nm, the six spectral curves of Hami melon overlap with pepper and zucchini, which is one of the reasons why Hami melon is easily confused with pepper and zucchini. In addition, Hami melon, pepper, and zucchini are all grown by strip cultivation in the study areas, and were in the same phenological period (fruit setting) when the experimental images were obtained, which weakens the differences in their texture features.

**Figure 10:**
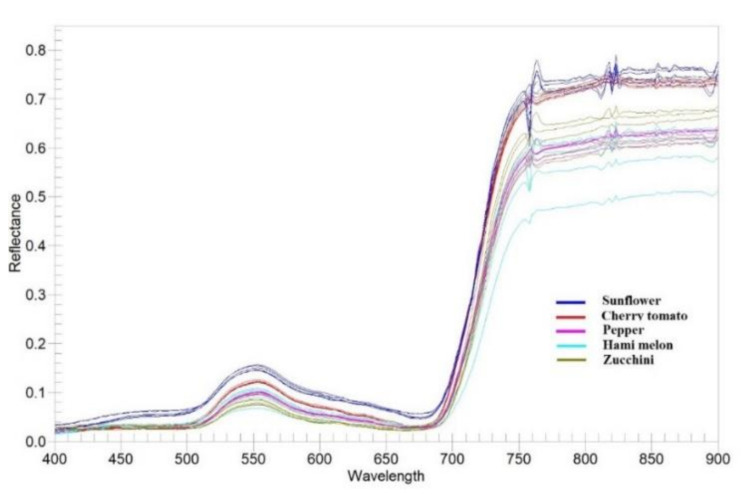
Spectral curve of ground objects.

Interestingly, although the reflectance of cherry tomato is obviously higher than that of Hami melon, and the reflectance of sunflower is obviously higher than that of zucchini in the near-infrared band of 770–800 nm, there are mixed fractions of Hami melon and cherry tomato, and mixed fractions of sunflower and zucchini, in SA3. One possible explanation is that the cherry tomato and sunflower are densely planted in the study area, with many overlapping leaves. Compared with single-leaf plants, multiple leaves can produce higher reflectivity in the near-infrared band due to additional reflectivity [47]. Therefore, cherry tomato and sunflower have a higher reflectivity than other crops in the near-infrared band. However, in Figure 10, the cherry tomato blossom in area m and the sunflower in area n grow poorly and are sparsely planted, decreasing their reflectivity in the green band of 540–560 nm and the near-infrared band of 770–800 nm. Thus, the difference between them is decreased. Moreover, Hami melon and cherry tomato are vine plants, which have similar textural features. The big leaves of sunflower and zucchini also weaken the differences in their respective textural features.

### 5.2. Model Performance under Different Planting Structure Complexity

The classification results for the three study areas were produced using the OB-RF and OB-SVM models. The overall accuracy values of SA1 given by OB-RF and OB-SVM are 97.085% and 99.126%, respectively. For SA2, the overall accuracy values are 92.610% and 99.078%, respectively, and for SA3 they are 88.994% and 97.207%, respectively. These results indicate that the OB-RF and OB-SVM classification accuracies decrease as the complexity of the planting structure increases. In particular, OB-RF’s overall accuracy decreased by 8.1%, while that of OB-SVM only decreased by 1.9%. In general, the advantage of OB-SVM’s classification accuracy becomes more prominent as the number of ground features increases. From the differences in the extraction accuracies of the different methods, OB-SVM’s extraction accuracy was obviously better than that of OB-RF in SA3.

The occurrence of classification errors in this study is related to the sample size limitation, such as for the saplings and Hami melon in SA3. Comparing area j with area o, and area m with area q, in Figure 10, it is clear that the classification error of OB-SVM is smaller than that of OB-RF in small sample areas. As a representative ensemble learning algorithm, the RF classifier achieved good results in the automatic extraction of remote sensing information [42,43]. However, the RF classifier is better suited to large samples and high-dimensional data, and thus requires a sufficient number of samples [44]. The SVM classifier specializes in analyzing small numbers of samples [39,45]. In this study, the number of features in the three test areas gradually increased, and the number of available training samples was minimal as the plots became more fragmented. The learning ability of the classifier from the training samples under this situation directly determined the accuracy of the classification results. Therefore, the classification accuracy of the OB-SVM method was superior to that of OB-RF in this study, because of the high sensitivity of the SVM classifier to the samples. These results indicate that the OB-SVM method is more suitable for the classification of crops in fragmented areas with highly complex planting structures.

### 5.3. Classification Potential of UAV Multispectral Remote Sensing Technology under Complex Planting Structures

The OB-SVM model achieved superior classification performance in extracting crop information in areas with low-, medium-, and high-complexity planting structures, based on UAV multispectral remote sensing. The overall accuracies of the three study areas were 99.13%, 99.08%, and 97.21%, and the extraction accuracy values were better than 92.59%, 94.81%, and 85.65%. As the planting structure complexity increased, the classification accuracy and extraction accuracy decreased, but the overall accuracy was only reduced by 1.92%. Using UAV visible light images, Park et al. [30] applied an object-oriented method to classify cabbage and radish, and obtained an accuracy of 84.7%. The overall accuracy reached 97.21%, even under a complex classification environment with eight different crops in this study. Chen et al. [48] pointed out that UAV visible light images led to lower interpretation accuracy than multispectral images in agricultural land classification. In addition, Ishida et al. [49] used UAV hyperspectral remote sensing technology to classify 14 ground objects with an overall accuracy of 94.00%, and compared with this, the classification results in this paper were not inferior.

It can be seen from the spectral curve of ground objects (Figure 9) that the most remarkable difference in the reflectivity of each crop was in the near-infrared band, which made a significant contribution to the classification. Additionally, the importance ranking of the multispectral bands (Table A1) suggests that the near-infrared band played an essential role in the classification results of each study area (importance ranked third, first and second for SA1, SA2, and SA3, respectively). The vegetation indices (DVI and RVI) obtained using the near-infrared band as the input variable, and the texture features obtained from the second-order matrix probability operation (near-infrared coordination, near-infrared information entropy, near-infrared correlation, near-infrared contrast, and near-infrared heterogeneity), also played essential roles in the classification results (Table A2 and Table A3). Thus, it can be concluded that the near-infrared band provides essential features that improve the extraction accuracy of the planting structure, and enable the fine classification of crops. This is the main advantage of multispectral remote sensing compared with visible light remote sensing. Besides this, multispectral remote sensing’s high price limits its applicability to agricultural production, although UAV hyperspectral remote sensing offers a higher spectral resolution than multispectral remote sensing. Indeed, multispectral remote sensing satisfies the requirements as far as crop classification is concerned. In general, multispectral remote sensing technology has a higher spectral resolution than visible light, and has higher cost performance than hyperspectral remote sensing. Thus, it offers a wider range of applications for the fine classification of farmland features under highly complex planting structures.

Based on UAV multispectral remote sensing images as data, we used the OB-SVM and OB-RF models to extract crops in areas with highly complex planting structures. We verified the application potential of this method in the extraction of complex planting structures. The conclusions can provide new ideas for obtaining accurate crop distribution maps in areas with complex planting structures, and technical support for stabilizing food security and protecting water resources.

## 6. Conclusions

This study has described the analysis and classification of multispectral images using UAV remote sensing technology. RFE was used to screen the spectral features and texture features of crops in the images, allowing the feature subsets of three study areas to be successfully constructed. The OB-RF and OB-SVM models were used for the fine classification of crops based on the above procedures. Field observations and visual interpretation were used to evaluate the accuracy of the classification results through the confusion matrix method. The main conclusions of this study are as follows:

(1) The OB-SVM model’s classification accuracy in areas with low-, medium-, and high-complexity planting structures was respectively 1.99%, 4.60%, and 8.22% higher than that of the OB-RF model. As the planting structure complexity increased, the classification advantages of the OB-SVM model became more evident. This indicates that the OB-SVM model offers higher classification accuracy under land fragmentation and highly complex planting structures, and is more suitable for the fine classification of farmland features with highly complex agricultural planting patterns;
(2) Based on UAV multispectral remote sensing technology and the OB-SVM classification model, the overall accuracy of the study areas with low, medium, and high complexity were as high as 99.13%, 99.08%, and 97.21%, respectively. The extraction accuracy of each crop was at least 92.59%, 94.81% and 85.65% in the three study areas, respectively. As the planting structure complexity increased from low to high, the classification accuracy and extraction accuracy decreased, but the overall accuracy only decreased by 1.92%. Therefore, UAV multispectral remote sensing technology has vast application potential for the fine classification of farmland features under highly complex planting structures. The conclusions can provide new ideas for accurately obtaining crop distribution maps in areas with complex planting structures, and then provide technical support for protecting food security and the rational allocation of water resources.

## Figures and Tables

**Table 1:** Main parameters of the unmanned aerial vehicle (UAV) and camera.

Unmanned Aerial Vehicle (UAV)		Camera	
Parameters	Values	Parameters	Values
Wheelbase/mm	900	Camera model	MicaSense RedEdge-M
Takeoff mass/kg	4.7–8.2	Pixels	1280 × 960
Payload/g	820	Band	5
Endurance time/min	20	Wavelength/nm	400–900
Digital communication distance/km	3	Focal length/mm	5.5
Battery power/(mAh)	16,000	Field of view/(°)	47.2
Cruising speed/(m·s^−1^)	5		

**Table 2:** Meteorological data of the study areas during the test.

	Air Temperature (°C)	Air Humidity (%)	Illuminance (1 × 10^4^ lux)	Wind Speed (m/s)	PM2.5 (μg/m^3^)	PM10 (μg/m^3^)
26 July 2020	25.43	67.08	23.28	2.20	15.00	16.50
29 July 2020	28.63	51.98	21.53	1.60	5.00	5.25
1 August 2020	28.65	58.13	23.48	1.50	13.00	14.00

**Table 3:** The specific parameters of FieldSpec Hand Held.

Parameters	Values
Spectral range	325–1075 nm
Spectral resolution	3.5 nm at 700 nm
Sampling interval	1.6 nm
Integration time	2^n^ × 17 ms for n = 0, 1, …, 15
Wavelength accuracy	±1 nm
Noise equivalent radiance	5.0 E–9 W/cm^2^/nm/sr at 700 nm

**Table 4:** Number of training and validation samples for each study area.

Study Area 1			Study Area 2			Study Area 3		
Crops	TS	VS	Crops	TS	VS	Crops	TS	VS
Corn	35	15	Corn	33	11	Sunflower	48	17
Sunflower	38	12	Sunflower	40	15	Zucchini	32	12
Zucchini	40	17	Zucchini	42	15	Hami melon	12	5
Bare land	20	8	Hami melon	25	8	Pepper	21	9
			Pepper	27	9	Sapling	12	6
			Bare land	18	6	Watermelon	14	6
						Cherry tomato	23	8
						Tomato	30	11
						Bare land	25	9

**Table 5:** Common vegetation indices.

Vegetation Indices	Full Name	Formula
NDVI	Normalized difference vegetation index	$\left(\rho_{NIR}-\rho_{R}\right)/\left(\rho_{NIR}+\rho_{R}\right)$
RVI	Ratio vegetation index	$\rho_{NIR}/\rho_{R}$
DVI	Difference vegetation index	$\rho_{NIR}-\rho_{R}$
ExG	Excess green	$2\rho_{NIR}-\rho_{R}-\rho_{B}$
VDVI	Visible-band difference vegetation index	$\left(2\rho_{G}-\rho_{R}-\rho_{B}\right)/\left(2\rho_{G}+\rho_{R}+\rho_{B}\right)$
NGBDI	Normalized green-blue difference index	$\left(\rho_{G}-\rho_{B}\right)/\left(\rho_{G}+\rho_{B}\right)$
NGRDI	Normalized green-red difference index	$\left(\rho_{G}-\rho_{R}\right)/\left(\rho_{G}+\rho_{R}\right)$
WI	Woebbecke index	$\left(\rho_{G}-\rho_{B}\right)/\left(\rho_{R}-\rho_{G}\right)$

**Table 6:** List of screening results of feature subsets.

	Feature Types	Feature Subset
SA1	Multispectral bands	Red-band; Blue-band
	Vegetation indices	VDVI; ExG
	Texture features	Red-mean; Green-mean; Blue-mean; Red-correlation; Green-homogeneity; Blue-correlation; NIR-correlation
SA2	Multispectral bands	Red-band; Green-band; Blue-band; NIR; Red-edge
	Vegetation indices	NGBDI; ExG; RVI;
	Texture features	Blue-contrast; Blue-entropy; Blue-homogeneity; Green-dissimilarity; Red-second-moment; NIR-dissimilarity
SA3	Multispectral bands	Red-band; Green-band; Blue-band; NIR; Red-edge
	Vegetation indices	NGBDI; RVI; DVI; NGRDI
	Texture features	Red-homogeneity; Green-homogeneity; Blue-dissimilarity; Red- correlation

**Table 7:** Accuracy evaluation of classification results for SA1.

Methods		Objects	Zucchini	Corn	Sunflower	Bare Land
	Accuracy (%)					
OB-RF	PA		98.31	100.00	95.19	98.01
	UA		90.90	99.83	99.30	93.98
	F		94.46	99.91	97.20	95.95
	Overall accuracy = 97.09%, Kappa = 0.95					
OB-SVM	PA		99.41	99.87	99.87	87.50
	UA		99.37	98.99	99.10	98.32
	F		99.39	99.43	99.48	92.59
	Overall accuracy = 99.13%, Kappa = 0.99					

**Note:** OB-RF stands for Object-oriented random forest classification model. OB-SVM stands for support vector machine classification model.

**Table 8:** Accuracy evaluation of classification results for SA2.

Methods		Objects	Zucchini	Corn	Sunflower	Bare Land	Pepper	Hami Melon
	Accuracy (%)							
OB-RF	PA		84.37	99.62	99.53	92.63	99.12	86.11
	UA		99.68	98.93	99.94	98.43	73.85	67.45
	F		91.39	99.27	99.73	95.44	84.86	75.65
	Overall accuracy = 92.61%, Kappa = 0.90							
OB-SVM	PA		99.74	99.69	99.57	91.07	96.48	99.51
	UA		99.40	97.61	99.41	98.87	98.53	99.29
	F		99.57	98.64	99.49	94.81	97.49	99.40
	Overall accuracy = 99.08%, Kappa = 0.99							

**Table 9:** Accuracy evaluation of classification results for SA3.

Methods		Objects	Zucchini	Sunflower	Bare Land	Pepper	Hami Melon	Watermelon	Tomato	Cherry Tomato	Sapling
	Accuracy (%)										
OB-RF	PA		93.33	90.55	91.77	89.01	98.35	100.00	99.94	72.04	26.67
	UA		88.12	95.39	89.32	79.41	80.32	88.24	85.69	98.85	72.73
	F		90.65	92.91	90.53	83.94	88.43	93.75	92.27	83.34	39.03
	overall accuracy = 88.99%, Kappa = 0.86										
OB-SVM	PA		98.87	99.15	92.07	93.59	98.35	100.00	99.94	91.84	84.22
	UA		99.98	99.68	94.08	94.19	80.32	98.47	91.51	98.82	87.13
	F		99.42	99.41	93.06	93.89	88.43	99.23	95.54	95.20	85.65
	Overall accuracy = 97.21%, Kappa = 0.97										

## Data Availability

The data are not publicly available due to [privacy].

## Appendix A

**Table A1:** The importance ranking of the multispectral bands.

Multispectral Bands	B1	B2	B3	B4	B5
SA1	Red band	Blue band	NIR	Green band	Red edge
SA2	NIR	Red edge	Green band	Red band	Blue band
SA3	Blue band	NIR	Red band	Red edge	Green band

**Table A2:** The importance ranking of the vegetation indices.

Vegetation Indices	B1	B2	B3	B4	B5	B6	B7
SA1	VDVI	ExG	DVI	RVI	NGBDI	NDVI	NGRDI
SA2	NGBDI	ExG	DVI	RVI	NGRDI	NDVI	VDVI
SA3	NGBDI	RVI	NDVI	ExG	DVI	VDVI	NGRDI

**Table A3:** The importance ranking of the texture features.

Texture Features	SA1	SA2	SA3
B1	Red mean	Red contrast	Red homogeneity
B2	Green mean	Blue entropy	Green homogeneity
B3	Blue mean	Blue homogeneity	Blue dissimilarity
B4	Red correlation	Green dissimilarity	Red correlation
B5	Green homogeneity	Red second moment	Red contrast
B6	Blue correlation	Near-infrared dissimilarity	Near-infrared contrast
B7	Near-infrared homogeneity	Near-infrared correlation	Near-infrared homogeneity
B8	Near-infrared entropy	Blue correlation	Blue correlation
B9	Red variance	Red dissimilarity	Red variance
B10	Green dissimilarity	Red entropy	Green correlation
B11	Blue entropy	Red-edge second moment	Near-infrared second moment
B12	Green entropy	Green homogeneity	Red-edge second moment
B13	Green second moment	Green correlation	Green dissimilarity
B14	Red-edge mean	Red-edge dissimilarity	Near-infrared contrast
B15	Red-edge homogeneity	Red-edge homogeneity	Red-edge homogeneity
B16	Near-infrared variance	Blue dissimilarity	Near-infrared dissimilarity
B17	Red contrast	Red variance	Red mean
B18	Blue variance	Green entropy	Green second moment
B19	Near-infrared contrast	Near-infrared variance	Near-infrared mean
B20	Green correlation	Red correlation	Green contrast
B21	Green contrast	Blue mean	Green mean
B22	Red dissimilarity	Blue second moment	Near-infrared entropy
B23	Red-edge variance	Near-infrared mean	Near-infrared correlation
B24	Near-infrared second moment	Near-infrared homogeneity	Blue mean
B25	Red homogeneity	Red mean	Red entropy
B26	Red entropy	Green contrast	Blue entropy
B27	Near-infrared dissimilarity	Red variance	Near-infrared variance
B28	Red second moment	Green mean	Green variance
B29	Green variance	Blue variance	Blue homogeneity
B30	Red-edge contrast	Red-edge contrast	Red-edge second moment
B31	Red entropy	Red-edge second moment	Red-edge correlation
B32	Near-infrared mean	Near-infrared entropy	Blue variance
B33	Red dissimilarity	Red homogeneity	Red dissimilarity
B34	Red contrast	Blue contrast	Near-infrared variance
B35	Near-infrared correlation	Near-infrared contrast	Red-edge homogeneity
B36	Blue homogeneity	Green second moment	Green entropy
B37	Blue dissimilarity	Green mean	Blue contrast
B38	Red-edge second moment	Red-edge correlation	Red-edge dissimilarity
B39	Red-edge correlation	Red-edge entropy	Red-edge entropy
B40	Blue second moment	Red-edge mean	Blue entropy
